# Advances in Self-Assembled Peptides as Drug Carriers

**DOI:** 10.3390/pharmaceutics15020482

**Published:** 2023-02-01

**Authors:** Yawen Gao, Lele Wang, Xue Zhang, Ziling Zhou, Xinzhu Shen, Haodong Hu, Rui Sun, Jihui Tang

**Affiliations:** Inflammation and Immune Mediated Diseases Laboratory of Anhui Province, Anhui Institute of Innovative Drugs, School of Pharmacy, Anhui Medical University, Hefei 230032, China

**Keywords:** peptides, self-assembly, nanostructures, influencing factors, drug carriers

## Abstract

In recent years, self-assembled peptide nanotechnology has attracted a great deal of attention for its ability to form various regular and ordered structures with diverse and practical functions. Self-assembled peptides can exist in different environments and are a kind of medical bio-regenerative material with unique structures. These materials have good biocompatibility and controllability and can form nanoparticles, nanofibers and hydrogels to perform specific morphological functions, which are widely used in biomedical and material science fields. In this paper, the properties of self-assembled peptides, their influencing factors and the nanostructures that they form are reviewed, and the applications of self-assembled peptides as drug carriers are highlighted. Finally, the prospects and challenges for developing self-assembled peptide nanomaterials are briefly discussed.

## 1. Introduction

Peptides are compounds composed of multiple amino acids linked by peptide bonds in a certain order. They are widely found in living organisms, have important biological functions and are the basis of life activities and biomaterial development. Compared with traditional biomedical materials, peptides have unique advantages, such as outstanding chemical diversity, non-toxic biodegradability and good biocompatibility, and are ideal biomedical materials. In recent decades, scholars have been inspired by their knowledge of amino acids, natural peptides and proteins, to construct self-assembled peptides [1]. In 1993, Zhang et al. accidentally designed and synthesized an ionic-complementary peptide, EAKl6, from yeast proteins that could self-assemble into nanofibers and thus form stable hydrogel membranes by complementary ion interactions [2]. In the same year, Ghadiri and colleagues designed a cyclic peptide consisting of eight amino acid residues. This cyclic peptide was self-assembled into a stacked conformation in an acidic aqueous solution by hydrogen bonding between side chains to form nanotubes [3]. Tjernberg et al. also reported a typical self-assembled peptide, KLVFF, with a sequence derived from Alzheimer’s-disease-associated β-amyloid [4]. In addition to this, several peptide sequences with self-assembly potential have been screened from natural proteins. Since then, self-assembled peptides have entered the public eye and become a hot research topic in biomedicine, materials chemistry and other scientific fields [5,6,7,8,9].

Peptide self-assembly is the formation of molecular aggregates with a specific order of arrangement by non-covalent forces using molecular recognition between molecules or between one fragment of a molecule and another fragment. Such non-covalent forces mainly include van der Waals forces, electrostatic interactions, hydrogen bonding and hydrophobic forces, which allow peptide assemblies to form special and ordered nanostructures [10]. Taking the study of diphenylalanine by Reches et al. as an example, they found that dipeptides with the sequence FF have good self-assembly ability and can form nanostructures with different morphological functions, such as nanotubes, nanoribbons, nanofibers and spherical vesicles [11]. Nowadays, nanotechnology has been extensively studied for the various biological properties of nanosystems that have been used for drug delivery, which is of great importance for the progress and development of biomedical science. It has been found that nano drug delivery systems have great potential to overcome barriers to cancer therapy [12,13]. These systems can not only facilitate drugs crossing the blood–brain barrier but also effectively improve the problem of drug resistance in chemotherapy, thus greatly improving therapeutic efficacy. However, when self-assembled peptide nanomaterials enter the complex physiological environment, the problems of how to improve the targeting of drug molecules and the stability of nanomaterial structures are still challenges to be faced. Although peptide molecules have problems, such as easy enzymatic disassembly and low drug loading and drug release efficiency, their good biocompatibility, excellent biodegradability, simple synthesis and easy preparation have shown important potential for their application as drug carriers [14,15,16].

This review provides an overview of the structure of self-assembled peptides and their related properties and discusses the factors affecting the formation of peptide self-assembly. In addition, we summarized the relevant research results of self-assembled peptides in recent years, focusing on the progress of research into their use as drug carriers.

## 2. Driving Force of Peptide Self-Assembly

**Figure 1:**
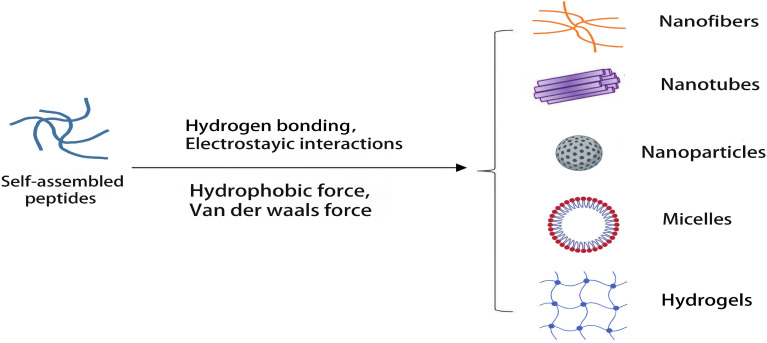
Nanostructures formed by self-assembled peptides.

The self-assembly of peptides is a spontaneous process driven by kinetics and thermodynamics. The formation of self-assembled peptide structures often results from the synergistic action of non-covalent interaction forces such as hydrogen bonds, hydrophobic interactions, π–π interactions and electrostatic interactions. Their interactions produce dynamic and responsive changes and form regular and complex polypeptide sequences and diverse nanostructures, as shown in Figure 1. Therefore, it is necessary to understand peptide self-assembly systematically through the types of non-covalent interactions.

### 2.1. Hydrogen Bonding

Hydrogen bonds is one of the important forces determining the peptide assembly structure and self-assembly process [17]. Hydrogen bonds can be formed between the amino and carboxyl groups on the main and side chains of a peptide. In this case, a plurality of peptide chains form the β-sheet secondary structure of the polypeptide by interacting with hydrogen bonds perpendicular to the adjacent peptide chains. Unlike the β-sheet structure, the α-helix is an intramolecular interaction because it is formed by hydrogen bonds between amide-based amino acids in the same peptide chain. Moreover, hydrogen bonds are selective and directional, are essential for the formation and stabilization of peptide secondary structures and can induce the assembly of peptides into different nanostructures [18].

### 2.2. Hydrophobic Interactions

Hydrophobic interactions are an important driving force for peptide self-assembly, and the thermodynamic driving force for most self-assembled structures is provided by the hydrophobic part. Hydrophobic interactions are stable in inducing peptide self-assembly, but hydrophobicity cannot provide directionality for peptide self-assembly alone [19]. It has been found that direction-independent hydrophobic interactions are one of the driving forces in surfactant systems [20]. When amphiphilic molecules are present in water, the hydrophobic portions of these molecules attempt to aggregate to minimize their surface area in contact with water, thereby exposing the hydrophilic portions to water. In addition, hydrophobic interactions can be significantly enhanced during salt-triggered self-assembly due to charge-shielding effects [21].

### 2.3. π–π Interactions

For many peptide molecules containing aromatic groups, π–π stacking is an important driving force for self-assembly. The π–π stacking can provide energy and directionality to the self-assembly process of peptides, thus inducing the directional growth of the assemblies. Because the solubility of peptides containing aromatic groups is generally poor, their self-assembled structures in aqueous solutions tend to be more stable [22]. It has been shown that the aromatic residues of peptide structural units can act through hydrophobic interactions or π–π interactions; in hydrophobic interactions, the accumulation of aromatic residues is usually disordered, but in π–π interactions, the accumulation of aromatic residues is ordered [23].

### 2.4. Electrostatic Interactions

In addition to the above-mentioned forces, another force that drives the self-assembly of peptides is the electrostatic interaction between the oppositely charged molecules of a peptide. Electrostatic interactions affect the self-assembly process through the charged groups of amino acids, there may be electrostatic attraction or repulsion and there are non-directional forces that are commonly used to induce structural specificity in charged peptides [24]. In addition, electrostatic interactions not only contribute to the embedding of drugs into self-assembled charged peptides but also allow the formation of highly stable and ordered nanostructures for various drug delivery systems [25].

## 3. Factors Affecting Peptide Self-Assembly

Peptides self-assemble mainly through the synergy of non-covalent forces between peptide molecules, and the assembly structure can be regulated by changes in external conditions. Meanwhile, the external environment, including pH, temperature, enzymes, ion concentration, solvents, etc., can affect or reverse the self-assembly process, as shown in Table 1.

### 3.1. pH

pH can affect the electrostatic and hydrophobic interactions of peptides and is an important factor in determining the structure of peptides. The amino acid side chains of peptides can show different charge tendencies at different pH conditions [26]. By changing the pH of the solution, the C- and N-terminal ends of the peptide chain, and some chemical groups, can be positively or negatively electrified. A peptide with the sequence ETATKAELLAKYEATHK has positively charged amino acids at the C-terminus and negatively charged amino acids at its N-terminal end. When the pH is 4, the peptide shows a clear α-helical structure. When the pH is 8, the α-helix structure of the peptide changes to a β-sheet-like structure [27]. Therefore, the secondary structure of the peptide can be changed due to pH fluctuations. In addition to this, Li et al. designed a biologically functional self-assembled peptide triggered by pH. The peptide was not only able to exert antibacterial effects but also induced bacterial aggregation and promoted effective phagocytic clearance at the site of bacterial infection. The results showed that the peptide could self-assemble into spherical micelles at a pH of 5. However, when the pH was adjusted to 6.0 and 7.0, the microstructure of the peptide changed, and the spherical micelles transformed into nanofibers, which could be transformed into longer nanofibers with increasing pH [28]. Therefore, by controlling pH, self-assembled peptides based on different pH levels can be designed rationally.

### 3.2. Temperature

Temperature is an important factor that, by changing hydrogen bonding and hydrophobic interactions, can lead to structural changes in peptide molecules. Peptides self-assembled by temperature changes initially exist mostly as monomers, but when the temperature increases, they change their morphology to form nanofibrils, micelles and other nanostructures such as diphenylalanine peptides [29], elastin-like peptides (ELPs), etc. It was found that ELPs exist as monomers below the transition temperature, but these monomers can be transformed into micelles under the action of thermal energy. Therefore, by heating ELP molecules, their hydrophobic activity increases, and they can form micellar structures in polar solvents [30]. Increasing the temperature can also break the intermolecular hydrogen bonds and enhance the hydrophobic interactions of peptides, thus affecting the stability of the self-assembled system. Tiné et al. designed a tetraionic peptide, RWDW, and found that temperature had a strong influence on its self-assembly process. At 15 °C and 25 °C, the tetrapeptide formed tight and intertwined fibers, and when the temperature increased to 35 °C, the fiber structure collapsed and the lines clustered further apart, which was in addition to the gradual weakening of the fiber layer seen at 25 °C. The results showed that RWDW differed in its morphology and depolymerization process at all three temperatures studied [31]. This indicates that changing the temperature in different systems can have different effects on the self-assembly behavior of the systems.

### 3.3. Enzymes

Enzyme-instructed self-assembly not only triggers the self-assembly of peptides but also controls the structure and morphology of self-assembled peptide nanomaterials [32]. Shi et al. found that the amphiphilic peptide, PP1, contains phosphorylated threonine, and dephosphorylation by phosphatase affects the folding of the peptide. When PP1 is dephosphorylated, the conformational equilibrium shifts toward a β-sheet conformation, which facilitates the formation of folded hairpin structures in self-assembled fiber networks. However, phosphorylated PP1 then forms a gel with increased stiffness, leading to fracturing of the fibril network [33]. This suggests that enzymes can be used as a tool to control the self-assembly of peptides and influence the construction of nanostructures. In addition, enzyme-instructed self-assembly also contributes to the self-assembly and hydrogenation of hydrophobic peptides. Wang and his colleagues analyzed two hydrophobic peptides, namely tyrosol peptide (YSV) and laminin pentapeptide (YIGSR), and found that these two peptides were unable to self-assemble into hydrogels due to their poor solubility. So, they designed phosphorylated precursors for both peptides, which have good solubility and can be dephosphorylated by alkaline phosphatase to form supramolecular hydrogels. This shows that enzyme-instructed self-assembly is an effective method for generating hydrophobic compound hydrogels and holds great promise for the preparation of hydrophobic nanomaterials [34].

### 3.4. Ion Concentration

Ion concentration has always been an important factor affecting peptide molecule stacking and the structure and function of proteins; the presence of salt ions causes the shielding of charged groups, which results in weaker electrostatic interactions between molecules. This charge shielding effect also causes an increase in intermolecular hydrophobic bonding forces, making peptide molecules more prone to polymerization, causing self-assembly [35]. Ozbas et al. studied the conformation of the hairpin molecule, MAX1, with and without salt ions. At the same peptide concentration (<2 wt%), temperature and pH (7.4), without salt, the peptide molecule showed a disordered structure. Upon the addition of a small amount of salt to the solution, the electrostatic interactions between the ions and the charged amino acids caused the MAX1 to rapidly form a β-hairpin structure and subsequently a β-folded structure. In addition, MAX1 can also self-assemble into a three-dimensional hydrogel network through hydrophobic interactions and intermolecular hydrogen bonding [21]. As a result of the role of multiple ions in regulating cellular metabolism, maintaining ionic balance inside and outside the blood vessels and promoting bone development in vivo, peptides self-assembled based on their ion concentration response have a wide application potential in the medical field.

### 3.5. Solvent

Solvents can influence and alter the morphology of peptide self-assembly and guide self-assembled peptide nanostructures to chiral inversion [36]. Huang et al. investigated the self-assembled morphology of diphenylalanine peptides in different solvents and found that they could easily achieve the structural transformation of self-assembled diphenylalanine peptides from microtubules to nanofibers in the aqueous phase by introducing acetonitrile as a co-solvent [37]. A similar situation was reported by Zhao and colleagues, where the self-assembly of an amphiphilic peptide (KI_4_K) was modulated by changing the solvent [38]. The results suggested that the solvent played an important role in guiding the self-assembly of KI_4_K into structures with different morphologies. In addition, Yang et al. synthesized four dipeptides derived from D- and L-alkanes with long alkyl chains and investigated their self-assembly behavior in water and tetrahydrofuran. It was shown that these dipeptides formed twisted nanobands and that terminal alanine chirality controlled the chirality of the nanobands. Interestingly, they formed nanoribbons with opposite habituations in both solvents [39].

## 4. Types of Self-Assembled Polypeptides

Self-assembled peptides are monomeric assemblies of short or repetitive amino acid sequences formed into nanostructured peptide assemblies that exhibit unique physicochemical and biochemical activities. Among them, peptides with secondary structures, amphiphilic peptides, ionic-complementary peptides and trans-stimulation-responsive peptides can self-assemble into different nanostructures. By considering the chemical properties of amino acids and peptides, nanostructures can be designed and synthesized from the following building blocks.

### 4.1. β-Sheet

**Figure 2:**
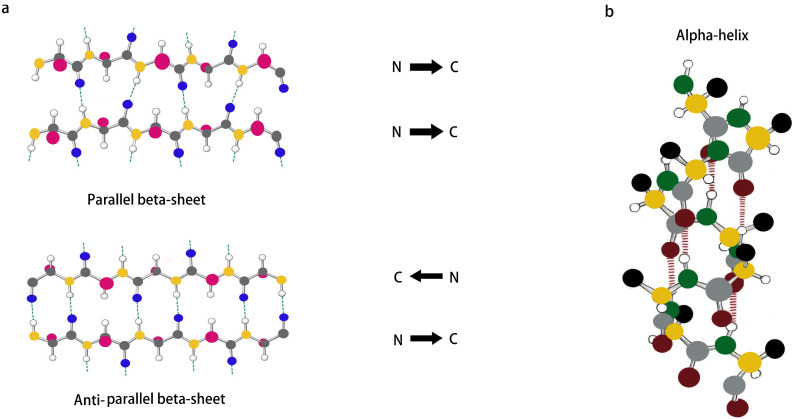
(**a**,**b**) Schematic representation of β-sheet and α-helix secondary structures.

Inspired by the sequence (Ac(AEAEAKAK)_2_-CONH_2_) found in zuotin, a Z-DNA-binding yeast peptide [2], many β-sheet structures have been formed, and many self-assembled peptide structures have already been designed. β-Sheets are flattened sheet-like structures formed by parallel or antiparallel arrangements of peptides that stabilize the structure of the peptide by interchain hydrogen bonding with neighboring main-chain amides, as shown in Figure 2a. Polypeptides forming β-sheets and supramolecular self-assembled structures usually contain 16–20 amino acids with an alternating distribution of polar and non-polar amino acids. In particular, the β-sheet structure separates the hydrophobic and hydrophilic regions of the peptide sequence, providing amphiphilicity to the peptide and driving the peptide self-assembly process. For example, a peptide comprising hydrophilic and hydrophobic amino acids was designed wherein the β-sheet was driven to grow axially into a protofibrillar nanostructure with a hydrophobic and hydrophilic surface, producing a bilayer structure that reduced the likelihood of the hydrophobic surface coming into contact with water [40,41]. β-hairpin peptides are derivatives of β-turns and usually consist of two hydrogen-bonded antiparallel β-chains bridged by reverse turns. Initially, Schneider et al. designed a class of MAX1 peptides consisting of alternating lysine and valine residues that could fold into a β-hairpin structure under the influence of external factors and then self-assemble into rigid hydrogels [42]. It has been shown that electrostatic interactions can also affect the formation of β-hairpin structures. By increasing the pH of the solution or the salt concentration of the solution to shield the electrostatic interactions, the peptides can form β-hairpin structures with hydrophilic lysine residues as the inner surface and hydrophobic valine residues as the outer surface, and they can then finally use their hydrophobic effect to self-assemble to form nanofibers [43,44,45].

### 4.2. α-Helix

The α-helix, which is usually formed by winding the peptide backbone into a right-handed helix containing 3.6 amino acids per turn, is an important secondary structure type in protein- and peptide-like structures and is also one of the main structures present inside a portion of peptide hydrogels. Unlike the structure of a β-sheet, an α-helix is formed by a single peptide chain in which the main-chain amide component is an intramolecular hydrogen bond, as shown in Figure 2b. Although α-helices are easy to synthesize and modify, linear peptides containing α-helical structures still lose their helical conformation in solution due to their inherent thermodynamic instability [46]. Therefore, stabilization of the α-helical structure is important for triggering the self-assembly of peptides [47,48,49]. It was found that side chain cross-coupling, hydrogen bonding substitution and the formation of salt bridges can be used to stabilize α-helical structures. Mihara et al. designed an α-helical structure with seventeen peptide segments, and they stabilized the α-helical structure using two sets of EK salt bridges and then further self-assembled it into a nanostructure [50]. In addition, Lee et al. showed that peptide self-assembly mediated by β-sheets can also be used to stabilize α-helix structures, which can enable peptides to self-assemble into nanostructures in solution [51]. The self-assembly of α-helices is achieved mainly by “helical coils”, which have good surface interactions between the helices and thus can generate helical beams [52]. Although most nanofiber assembly comes from β-sheet structures, nanofibrils and other nanostructures can also be formed from α-helical polypeptides by hydrophobic segment modification [53] and the lateral binding of multiple helical coils [54,55].

### 4.3. Surfactant-Like Peptides

In general, surfactant-like peptides are composed of a hydrophilic head attached to a hydrophobic tail. The hydrophilic head generally consists of one or two charged amino acid residues (His, Asp, Glu), and the hydrophobic tail generally consists of between three and nine nonpolar amino acids (Ala, Phe, Ile, Val). When dispersed in aqueous solutions, they tend to self-assemble and hide their hydrophobic tails through polar interfaces, a behavior that enables the formation of nanostructures such as nanotubes or nanocapsules [56]. Nanotubes or nanocapsules become the main structures formed by the assembly of surfactant-like peptides through self-assembly, and they can act on cellular lipid bilayers in a similar way to lipid-scavenging micelles [57,58]. There is also a specific peptide, called bola-amphiphilic peptide, that exhibits a different linkage, where a hydrophobic sequence located at the center can be linked to charged amino acids at both ends [59]. The difference between bola-amphiphilic peptides and surfactant-like peptides is the number of hydrophilic head groups on the building blocks: surfactant-like peptides have only one head, whereas bola-amphiphilic peptides have two hydrophilic heads with two hydrophilic groups spaced by hydrophobic sites at both ends [60,61]. Given their structural similarities, bola-amphiphilic peptides can display surfactant-like peptide properties. It has been shown that the two heads of bola-amphiphilic peptides are usually positively charged amino acids (KAAAAK, KAAAAAAK and RAAAAAR) capable of binding to negative residues of nucleotides that are often used to bind DNA/RNA [62].

### 4.4. Amphiphilic Peptides

The structure of amphiphilic peptides is similar to that of lipid molecules and is most typically characterized by the attachment of hydrophobic long-chain alkyl groups to hydrophilic peptides. The hydrophilic head and hydrophobic tail of amphiphilic peptides can interact with each other to stabilize various supramolecular structures by inter- and intramolecular driving forces, and they can also generate nanostructures with different morphologies [63,64,65]. As novel self-assembled peptide nanomaterials, amphiphilic peptides are of interest because of their simplicity, versatility and biocompatibility. In terms of their biomedical applications, amphiphilic peptides are easy to design and synthesize, thus ensuring their quality and purity. Moreover, nanostructures generated from amphiphilic peptides can show high biological activity and play an important role in tissue engineering, regenerative medicine and drug delivery [66]. Amphiphilic peptides are commonly used as carriers for the delivery of antitumor and nucleic acid drugs to their targets and have also been used to transport some therapeutic peptides. Cirillo et al. designed an amphiphilic peptide, G(IIKK)_3_I-NH_2_ (G3), for applications in targeted drug delivery. It was found that the amphipathic peptide, G3, not only has a high affinity for colon cancer cells but also has an endosomal escape ability. When the targeted siRNAs ECT2 and PLK1 were used, G3 was able to carry nucleic acids across cell membranes and successfully deliver them both to cancer cells. G3 not only has good anti-cancer activity and specificity but can also act as a gene carrier to regulate gene expression in cancer cells, which makes G3 promising as an excellent cancer therapeutic agent [67].

### 4.5. Ionic-Complementary Peptides

Ionic-complementary polypeptides are composed of alternating arrangements of hydrophilic and hydrophobic charged amino acids that can self-assemble into nanofibrous hydrogels at the molecular level. Ionic-complementary polypeptides have a unique charge distribution pattern and can be classified into three types: Type I (−+−+−+−+), Type II (−−++−−++) and Type IV (−−−−++++). Depending on their charge distribution order, they undergo self-assembly in different ways, and ionic complementary peptides can be designed rationally by repeating and combining charge distributions. At present, in addition to the initially discovered self-assembled peptide EAK16, many ionic complementarity peptides with unique advantages, such as RADA16-I and KFE8, have also been widely studied in various fields. Among them, RADA16-I, as a classical ionic complementarity peptide, has been used in biomedical and clinical fields for its ability to spontaneously form fibrous hydrogels in aqueous solutions [68]. Nevertheless, at the same time, RADA16-I exposes the common problem of most of these peptides, namely, their instability at low pH. To further consolidate the use of ionic-complementary peptides for medical applications, scientists have worked on “modifying” these peptides. Moreover, to better refine the advantages of ionic-complementary peptides, it has recently been shown that hydrogels driven by two complementary ionic peptides with opposite charges exhibit better biocompatibility with fibroblasts at physiological pH levels, once again demonstrating the potential of hydrogels for biomedical applications [69].

### 4.6. Chemical-Group-Modified Peptides

**Figure 3:**
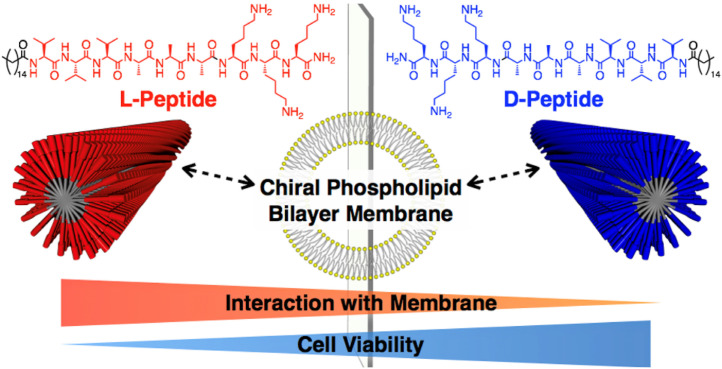
Chiral recognition of lipid bilayer membranes by supramolecular assemblies of peptide amphiphiles. Reprinted with permission from ref. [71]. Copyright (2019) American Chemical Society.

It was found that the self-assembly of peptides can be promoted by covalently binding chemical groups with self-assembly functions to peptide molecules, altering the non-covalent interactions of the peptides. Chemical-group-modified peptides exhibit an increased secondary structure, thereby making the nanobody more stable [70]. Chemical groups can play specific roles by designing corresponding functional regions in the peptide chain. Currently, linkage to hydrophobic alkyl chains is the most common modification in peptide building blocks. Hydrophobic forces, as the central force driving the self-assembly of peptides, can modify the properties and functions of peptides by designing linkages to alkyl peptide chains at the amino terminus. Sato et al. investigated the effect of peptide chirality on their cytotoxicity and cell membrane binding ability by using alkylated, modified D and L-type-V_3_A_3_K_3_ assembled peptides [71], as shown in Figure 3. In addition to hydrophobic alkyl chains, lipid groups and sugars can also be used to modify peptides. Sugars are involved in various life activities in the body. For example, mannose can target macrophages, while the hydroxyl group on the sugar greatly increases the hydrophilicity of the whole molecule. Xu et al. designed and synthesized an enzyme-responsive glycopeptide derivative that could be sheared by β-galactosidase in senescent cells and self-assemble into gels. The gels further induce apoptosis in senescent cells and act as clearing agents for these cells [72].

### 4.7. Metal-Coordination Peptides

Metal-coordination peptides, using peptides and metal ions as building blocks, combine the advantages of peptide self-assembly and metal–ligand interactions and have good prospects for the construction of novel nanomedicines [73]. For example, histidine-rich peptides can form coordination interactions with metal ions and further self-assemble into functional supramolecular nanomaterials, which play an essential role in optoelectronic engineering [74], bio-nanotechnology [75], drug delivery and biomedicine [76]. It has been found that metal ligands can control the self-assembly behavior of peptides, which can produce different morphologies depending on the binding stoichiometry and geometry of the metal ions. Knight et al. synthesized a peptide–polymer amphiphile, oSt(His)_6_, which was observed to have different morphological structures in different divalent transition metal ions (Mn^2+^, Co^2+^, Ni^2+^, Cu^2+^, Zn^2+^ and Cd^2+^). Aggregated micelles were observed in the presence of Zn^2+^, Co^2+^ and Cu^2+^, isolated micelles were observed in the orientation of Ni^2+^ and Cd^2+^, and multilayer vesicles were formed when oSt(His)_6_ was coordinated to Mn^2+^ [77]. This work demonstrated the great potential of transition metal ion coordination as a tool to guide the assembly of synthetic nanomaterials. Furthermore, peptides can also be used as templates to bind to metal surfaces to modulate metallic nanomaterials with different shapes, structures and compositions. Feng et al. used the self-assembly properties of the peptide Aβ_25-35_ to assemble and prepare monomers, protofibrils and mature fibers by incubating them in phosphate buffer and water for different times. Au nanoparticles, Au nanoribbons and Au nanofibers were then successfully synthesized based on these three self-assembled structures, respectively, and their catalytic activities were confirmed to be significantly higher compared to Au nanoparticles prepared without a template [78]. Thus, changes in self-assembly conditions induce differences in the self-assembled structures of peptides, which can lead to the formation of many different structures of Au nanoparticles.

### 4.8. Stimulus-Responsive Peptides

Amino acids are linked by amide bonds to produce a large number of polypeptides of different lengths and sequences, and the nature of the amino acids themselves and the order in which they are arranged in the polypeptide chain can influence the self-assembly behavior of the polypeptides. It has been found that the integration of stimuli-responsive amino acids into polypeptides can modulate the non-covalent interactions between polypeptides, thus controlling their conformation and assembly propensity. This strategy has been widely used to design stimulus-responsive peptide assembly modules, including redox-responsive peptides, pH-responsive peptides, light-responsive peptides and enzyme-responsive peptides [79]. Singh et al. designed arginine-α,β-dehydroxyphenylalanine dipeptide nanoparticles (RΔF-NPs) by encapsulating adriamycin in RΔF-NPs to form RΔF-Dox-NPs. These consisted of Dox as a functional module and RΔF-NPs as a response module. In the tumor microenvironment, RΔF-Dox-NPs have a higher drug release, an enhanced cancer cell internalization and an enhanced cytotoxicity at acidic pH, and they can serve as more effective pH-responsive drug delivery systems [80]. In recent years, enzyme-responsive drug delivery systems have also received much attention from researchers. Enzymes are highly selective biocatalysts that play a key role in many biological processes and in a variety of disease processes. Peptides are important bioactive substances in living organisms, and specific peptide sequences can be hydrolyzed by specific proteases, offering the possibility of constructing enzyme-responsive peptide carriers. Song et al. self-assembled a photosensitizer, PpIX, and an immune checkpoint inhibitor, 1-methyltryptophan, by linking them through the apoptosis enzyme-sensitive sequence DEVD to form uniformly sized nanomicelles, which were aggregated and retained for a long time in vivo by the EPR effect in the tumor region. Light exposure to the tumor area triggered the production of reactive oxygen species by the photosensitizer PpIX to induce apoptosis, which in turn generated apoptotic enzymes and promoted the release of tumor cell antigens [81]. In addition, the generated apoptotic enzymes hydrolyzed the enzyme-sensitive sequence DEVD, which induced the detachment of 1-methyltryptophan from the nanoparticles and subsequently activated an in vivo immune response for the inhibition and clearance of tumor lesions.

Polypeptides can self-assemble into a variety of nanostructures not only under endogenous stimuli such as pH, enzymes, etc. [82], but also by using external physical stimuli such as light, ultrasound, magnetic fields, etc. Li et al. designed a multi-component ligand self-assembly strategy based on combining peptides, photosensitizers and metal ions to construct metallo-nanodrugs for antitumor therapy. Under the action of metal ions, peptides and photosensitizers can perform synergistic self-assembly to form metallo-nanodrugs. Compared with photosensitizers alone and conventional drug delivery systems, metallo-nanodrugs can integrate robust blood circulation. They can target burst release, which can prolong blood circulation, increase tumor aggregation and enhance the antitumor effects of photodynamic therapy [83]. Ultrasound can also be used as a stimulation trigger for drug delivery systems, which are non-ionizing and non-invasive, have deep tissue penetration and can safely transmit acoustic energy to the appropriate local area. Sun et al. first co-assembled polylysine with Pluronic F127, which was chemically cross-linked to form a stiffness-adjustable nanogel (GenPLPF). Subsequently, they added the targeting agent ICAM-1 antibody and the chemotherapeutic agent epirubicin (GenPLPF_T_/EPI) to the nanogel to target tumors and enhance the anti-tumor ability actively. It was found that ultrasound modulated the deformability and stiffness of GenPLPF_T_/EPI, achieving a balance between prolonged blood circulation and deep tumor penetration, allowing more drugs to be accumulated in the tumor tissue. In addition, ultrasound can reduce the interstitial pressure of tumor tissue, helping to promote blood flow and more profound tumor enrichment with efficient anti-tumor effects [84].

## 5. Self-Assembled Peptide Nanostructures and Their Application to Drug Carriers

Owing to the designability of peptide modules and their adjustable physicochemical properties, self-assembled peptide nanomaterials can be easily constructed into various structures and shapes, including nanoparticles, nanofibers, micelles, hydrogels, etc., as shown in Table 2. Self-assembled peptide nanostructures are good candidates for effective drug carriers due to them having many advantages such as high drug loading efficiency, low drug loss rate, high stability and avoiding in vivo clearance [85]. The hydrophobic groups of self-assembled peptides can allow the drug or the peptide itself to penetrate the cell membrane, thus improving the bioavailability of the active ingredient. In contrast, the ligand–receptor interactions on the surface of the peptide can target the active ingredient to a specific site.

### 5.1. Nanofibers

Generally, the diameter of nanofibers is less than 100 nm. Studies have shown that hydrophobic collapse and β-sheet structures lead to the formation of nanostructures, including ribbon or cylindrical nanofibers [63]. β-Sheet-structure-mediated self-assembled peptides can stabilize α-helices, thus facilitating the formation of nanostructures of self-assembled peptides in aqueous environments [51]. Natural peptides, such as EAK16, RADA16 and elastic-like peptides, can be folded into β-sheet structures and subsequently self-assembled into nanofibers [100,101]. Yokoi et al. reported that RADA16-I peptides, composed of aspartic acid, alanine and arginine, were designed to generate regular β-sheet structures and self-assemble to form nanofibers [86]. In addition, amphiphilic peptides with alkyl groups are the most commonly used peptides for the formation of nanofibers. Because of their amphiphilic surfactant structural characteristics, amphiphilic peptides can self-assemble into nanostructures in water through hydrophobic interactions and function as bioactive peptides. Zhang et al. combined a tau-protein-derived peptide with the antitumor drug camptothecin, and this amphiphilic complex could form stable nanofibers or nanotubes through hydrophobic interactions and intermolecular hydrogen bonding, which effectively improved the solubility and selectivity of camptothecin, enhancing its medicinal effects [87]. Similarly, Ding et al. reported a self-assembled peptide nanostructure that could encapsulate 10-hydroxycamptothecin (HCPT), which exhibited a sustained and slow release and better antitumor activity in vivo compared to free HCPT [88].

### 5.2. Nanotubes

**Figure 4:**
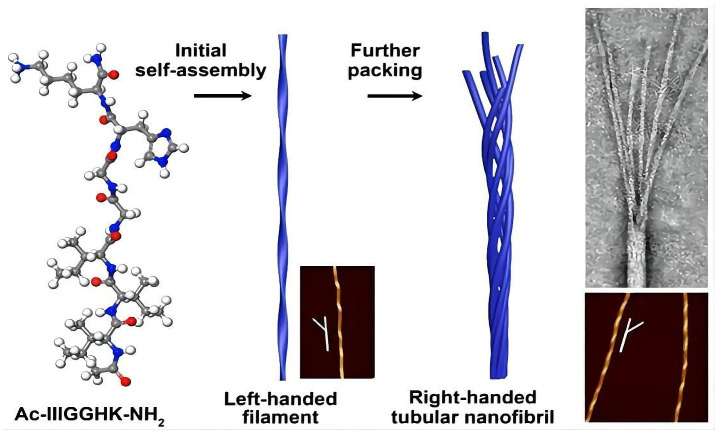
The self-assembly process of the amphipathic short peptide Ac-I_3_GGHK-NH_2_. Reprinted with permission from ref. [91]. Copyright (2021) American Chemical Society.

Nanotubes are elongated nanostructures that are similar to the structure of nanofibers. Cyclic peptides are the most commonly used nanotube materials. They have high stability compared to other peptide building blocks due to their unique properties, including increased drug loading, environmental stability, enhanced permeability and modifiable drug release. These hollow tubular structures can be composed of peptides with different amino acid sequences and enantiomers. They can be used for antiviral and antibacterial drug delivery and as gene delivery vectors [102]. For example, Zhang et al. designed an eight-residue cyclic peptide that was capable of self-assembling into cyclic-peptide-based nanotubes (CPNTs) in microscale aggregates. They loaded CPNTs with adriamycin (DOX) and further modified them using polyethylene glycol (PEG). Compared to free DOX, the polyethylene glycol-modified DOX-loaded CPNTs exhibited a higher cytotoxicity in human breast cancer MCF-7/ADR cells in vitro, altering the intracellular distribution of DOX and increasing the uptake of DOX [89]. Cyclic peptide nanotubes constructed with an even number of alternating D- and L-α-amino acids have been reported to exhibit antiviral activity. Hore et al. found an eight-residue cyclic D- and L-α-peptide with anti-influenza-A-virus activity that acted by preventing HeLa cells from forming low-pH endocytic carriers and further inhibiting virus escape from endosomes without adversely affecting cell viability [90]. In addition to cyclic peptide nanotubes, amphiphilic and surfactant-like peptides can also form nanotubes by self-assembly [103,104]. By investigating the self-assembly process of the amphiphilic short peptide Ac-I_3_GGHK-NH_2_, Zhao et al. found that it first assembled into left-handed nanofibers and then packed sequentially into right-handed nanotubes [91], as shown in Figure 4.

### 5.3. Nanoparticles

**Figure 5:**
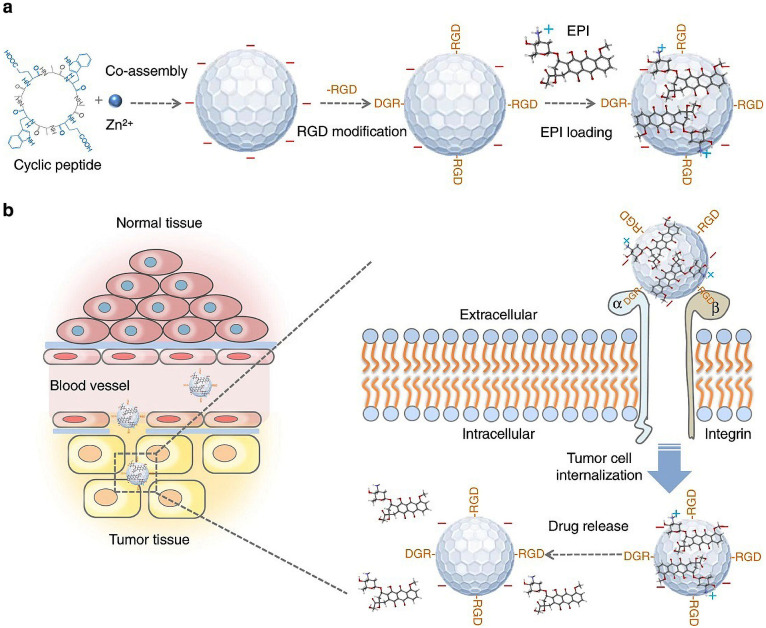
A schematic illustration of the synthesis of RGD-f-PNPs/EPI and its targeted EPI delivery into EC cells. (**a**) The RGD-f-PNPs were first synthesized by co-assembly of Zn2+ ions and cyclo[-(d-Ala-L-Glu-D-Ala-L-Trp)2-] peptides and were then modified with RGD peptide moieties onto the surface of f-PNPs. The EPI was loaded with RGD-f-PNPs through π–π stacking and electrostatic interactions. (**b**) The EPI-loaded RGD-f-PNPs can be used for targeted imaging and destruction of EC cells due to their capability of actively targeting and their enhanced penetration. Specifically, the RGD-f-PNPs/EPI nanoconjugates tend to accumulate more in the tumor tissue compared to normal tissues due to the enhanced permission and retention (EPR effects). Moreover, RGD peptide moieties bind to the overexpressed αvβ3 integrin subunits and internalize into EC cells. The embedded EPI is released from the RGD-f-PNPs and eventually accumulates in the nucleus to kill the cancer cells. Reprinted with permission from ref. [92]. Copyright (2018) the author(s).

Nanoparticles can range from nanospheres with hollow cores to a wide variety of solid structures composed of different building blocks [105]. For example, there is a self-assembled host defense protein-derived peptide, in the form of a β-sheet and a β-turn, which self-assembles to form nanoparticles that can exhibit better antibacterial effects [106]. Fan and colleagues modified fluorescent nanoparticles (f-PNPs) assembled from cyclic peptides with RGD peptides and then implanted epirubicin (EPI) into the nanoparticles to form a nanocouple (RGD-f-PNPs/EPI). RGD-f-PNPs/EPI can directly target the a_v_β_3_ integrin receptor in esophageal cancer for chemotherapy and can generate visible and near-infrared fluorescence for monitoring drug delivery to the tumor site and therapeutic response, as shown in Figure 5. RGD-f-PNPs/EPI not only enables in vivo targeted tumor imaging and EPI delivery but also reduces the risk of tumor damage compared to free EPI, reduces EPI-induced cardiotoxicity and improves therapeutic efficacy compared to free EPI [92]. Nanoparticle formulations of drugs can improve drug absorption and thus drug efficacy, require a lower drug dose and have fewer side effects than other standard formulations. Furthermore, nanoparticles can also help a drug to cross the biological barriers in the body and deliver the drug to the target organ, for example, across the blood–brain barrier to the brain. The blood–brain barrier is one of the most challenging biological membranes encountered in drug delivery, preventing not only the entry of harmful substances into the brain but also the entry of therapeutic drugs. With the development of self-assembled peptides, researchers have assembled a cellular-membrane-penetrating peptide, TAT, and coupled TAT peptides with amphoteric ionic nanogel to form TAT-nBSA nanoparticles, which have been confirmed to cross the blood–brain barrier [93]. In addition, peptides designed as nanoparticles for targeting cancer cell surfaces or tumor vasculature in chemotherapy can minimize systemic adverse drug reactions and improve efficiency. Zhao et al. synthesized PEG-Hb-PTX NPs loaded with paclitaxel (PTX) by acid denaturation using polyethylene-glycosylated hemoglobin (PEG-Hb) as a drug carrier. It was found that the PEG-Hb-PTX NPs could accumulate in tumor tissues in large quantities and exhibited excellent anticancer activity. Compared with conventional paclitaxel preparations, PEG-Hb-PTX NPs have better in vivo antitumor effects [94].

### 5.4. Micelles

**Figure 6:**
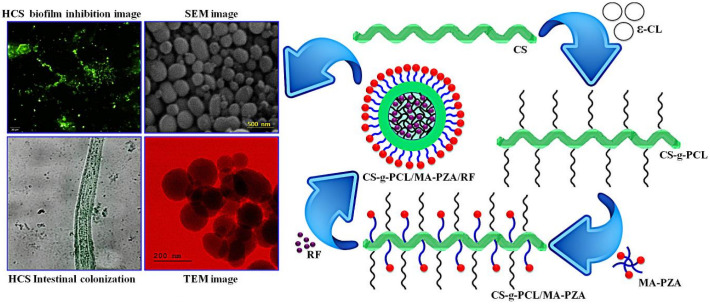
Schematic illustration for the preparation, characterization and application of CS-g-PCL/MA-PZA/RF micelles. Reprinted with permission from ref. [95]. Copyright (2020) Elsevier.

A micelle is a special conjugate with a hydrophobic core and a hydrophilic outer layer. Amphiphilic polymer molecules, obtained by linking hydrophobic fragments to hydrophilic polymers, can form micelles and can be used for the loading and delivery of insoluble molecules. Polymer micelles are mainly made of diblock polymers, triblock polymers or grafted polymers with hydrophilic and hydrophobic portions. For example, charged amphiphilic block copolypeptides [poly(L-lysine)-b-poly(L-leucine)] self-assemble in an aqueous solution to form stable vesicles and micelles [107]. Among other things, their hydrophobicity contributes to their rigidity and stability. In recent years, polymeric micelles have been widely used for drug delivery because of their robust core–shell structure, kinetic stability and inherent solubility properties. Shi et al. synthesized chitosan-grafted polycaprolactone/maleic anhydride pyrazinamide (CS-g-PCL/MA-PZA) polymers that can be used for the delivery of anti-methicillin-resistant-Staphylococcus-aureus drugs such as rifampin and pyrazinamide, as shown in Figure 6. The prolonged release time and increased release rate of rifampicin and pyrazinamide in polymeric micelles provided better antimicrobial efficacy compared to other formulations [95]. Similarly, Bao et al. designed a bifunctional peptide that generated polymeric micelles (Pep-PEG-PCL/Ce6) by the surface modification of polyethylene glycol-b-polycaprolactone (PEG-PCL) micelles. The peptide conferred antiphagocytic and matrix metalloproteinase-2 cleavable properties to the micelles, enhancing the accumulation of Pep-PEG-PCL/Ce6 micelles in tumors in vivo and thus improving tumor suppression in photodynamic therapy. Compared to pristine micelles, micelles modified with bifunctional peptides produced significant anticancer activity [96].

### 5.5. Hydrogels

**Figure 7:**
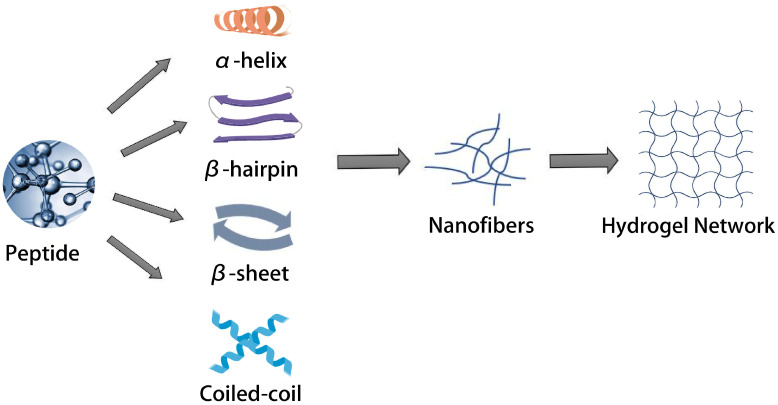
Simplified schematic illustrations of the hierarchical self-assembly process involved in the formation of hydrogels from peptides.

A hydrogel is a polymeric network system with a hydrophilic, three-dimensional mesh-like cross-linked structure. The formation of self-assembled peptide hydrogels is a segmented process [108], as shown in Figure 7. The peptide molecules in solution first form special secondary structures such as α-helix, β-fold, β-hairpin or curly helix, and then, after a certain degree of external stimulation or appropriate physical conditions, they self-assemble to form nanofibers; with an increase in time or concentration, the nanofibers in three-dimensional space gradually become thicker and longer, thus forming the structure of a fiber network. Water molecules are wrapped in the network structure of the peptide, and a hydrogel with certain mechanical strength is formed macroscopically. The properties of hydrogels formed by self-assembled peptides depend on pH, ionic strength and temperature [109]. It was found that the simplest dipeptide building block, diphenylalanine (Fmoc-ff) modified by Fmoc, can form hydrogels consisting of a network of nanofibers in an aqueous solution [110]. By modifying these peptides to Fmoc-FRGD and Fmoc-RGDF, it was found that the inclusion of RGD motifs also produced hydrogel structures but was unstable above pH 6.5.

Hydrogels have specific physicochemical and biological properties, and their hydrophilicity, flexibility and versatility also make them an excellent choice for drug delivery carriers in cancer therapy [111]. Jin et al. synthesized a novel bee venom peptide-RADA_32_-indocyanine green (ICG) hydrogel (MRI hydrogel) containing bee venom peptide in the peptide hydrogel backbone and ICG in the hydrogel matrix. It was shown that MRI hydrogels carrying bee toxins and ICG-based photothermal therapy had a synergistic effect and an enhanced photothermal treatment of glioblastoma [97]. Similarly, Zhang and colleagues incorporated indocyanine green and the immunostimulant polyinosinic:polycytidylic acid (poly I:C) into alginate/collagen to make an injectable thermo-responsive hydrogel (pTRG). Studies have shown that TRG plays a dual anti-cancer and prevention-of-cancer-metastasis role by combining photothermal therapy (PTT) and immunotherapy. It can not only eliminate tumors by PTT but also be used as a carrier of cancer therapeutic drugs, especially immunotherapeutic reagents, which presents good prospects for its application in the field of drug delivery [98]. Furthermore, hydrogel systems with a high water content are also widely used in tissue engineering because the large specific surface area of hydrogels creates a better environment for cell proliferation and differentiation. Meanwhile, the network structure inside the gel can allow the passage of some small nutrient or metabolite molecules, making it similar to the microenvironment in which normal cells live. It has been reported that wrapping D-RADA16 peptide hydrogel around nano artificial bone incorporating basic fibroblast growth factor (bFGF) not only reduced the degradation rate of the hydrogel but also prolonged the slow release of bFGF, thus better promoting bone healing and maintaining good bone healing properties [99]. In addition, RADA hydrogels can promote neutrophil growth and rat pluripotent stem cell differentiation by providing an extracellular matrix-like environment [112,113]. Self-assembled synthetic hydrogels of RADA16 peptides injected into a mouse model were observed to have a structure that facilitated chondrocyte embedding and articular cartilage regeneration and were accompanied by a lower inflammatory response than non-biological gels. This suggests that self-assembled peptide hydrogels can be used as cell culture scaffolds for tissue regeneration [114].

## 6. Summary

In this paper, we reviewed the progress of the research on self-assembled peptides as drug carriers in terms of their structure, properties and applications. Self-assembled peptides are considered novel agents with molecules composed of natural amino acids, which have high biocompatibility and low toxicity and thus can avoid the side effects associated with drugs; the molecules are highly designable and can self-assemble to form rich nanostructures of various morphologies and sizes, so their potential to function as carriers is high. They can form aggregates with defined structures under specific conditions and have molecular structure stability. They are responsive to stimuli, which is conducive to modulating their self-assembly process and the loading and release of functional components through changes in external conditions, e.g., for the controlled release of drugs. In addition, due to their inherent physicochemical properties, they can be loaded with both hydrophobic and hydrophilic drugs, which makes them suitable for triggered drug delivery at disease sites by inserting the stimulus-responsive part of their structure. In addition, peptide self-assembly can generate a series of complex and ordered nanostructures, which have important prospects for applications in biomedical fields such as drug delivery, tissue engineering, etc.

Self-assembled peptides are widely found in nature and are involved in regulating various life activities, but there are some problems in using self-assembled peptides as drug carriers. For example, self-assembled peptides as scaffolding materials for in vivo drug release are susceptible to the physiological environment, and their self-assembly is not stable enough. Although peptides can be assembled in various forms, the direct prediction and precise regulation of peptides from their molecular structures are still challenges. The research on self-assembled peptide nanomaterials needs to be more comprehensive, and the synthesis and application of most self-assembled peptide nanomaterials are still subject to some limitations. Their mechanical properties and functional characteristics still need further improvement. In the future, more work should be carried out to study the self-assembly process of peptides, rationalize the design of self-assembled peptide nanostructures and widely explore the application of self-assembled peptide nanomaterials as drug carriers. In addition, self-assembled peptides should be integrated with more functionalities when used as drug carriers, such as targeting ability, sensitivity and direct therapeutic effects. These difficulties and challenges will drive the development of the field, and the future of peptide-based self-assembled nanomaterials is promising and achievable.

## Figures and Tables

**Table 1:** Target sites of external factors on self-assembled peptides.

External Influencing Factors	Target Sites
pH	Charged amino acids, hydrophobic bond
Temperature	Hydrogen bond, hydrophobic bond
Enzymes	Secondary structure
Ionic concentration	Hydrophobic bond, electrostatic force
Solvent	Chiral structure

**Table 2:** Some nanostructures of self-assembled polypeptides and their applications.

Category	Polypeptide Module	Applications	Refs
Nanofibers	β-Sheet, amphiphilic peptides	Drug delivery	[86,87,88]
Nanotubes	Cyclic peptide, amphiphilic peptides, surfactant-like peptides	Drug delivery, drug stabilization	[89,90,91]
Nanoparticles	β-Sheet, amphiphilic peptides, surfactant-like peptides	Drug delivery, drug targeting	[92,93,94]
Micelles	Amhiphilic polymer	Drug delivery	[95,96]
Hydrogels	Amphiphilic peptides, surfactant-like peptides, ionic complementary peptides	Drug delivery, tissue engineering	[97,98,99]

## Data Availability

Not applicable.
